# Antioxidant, Anti-inflammatory and Antiproliferative Effects of Aqueous Extracts of Three Mediterranean Brown Seaweeds of the Genus *Cystoseira*

**Published:** 2014

**Authors:** Lamia Mhadhebi, Amel Mhadhebi, Jacques Robert, Abderrahman Bouraoui

**Affiliations:** a *Unit of Research of Bioactive Marine Substances (URSAM), Laboratory of Pharmacology, Faculty of Pharmacy of Monastir, Street Avicenne, 5000 Monastir, Tunisia. *; b *Laboratory of Pharmacology , University Victor Segalen Bordeaux 2, Institut Bergonié, 229 Street of Argonne, 33076 Bordeaux Cedex, France.*; c *Laboratory of Chemistry, Faculty of Sciences of Monastir, Street of Environement, 5000 Monastir, Tunisia.*

**Keywords:** Antioxidant activity, Antiproliferative activity, Anti-inflammatory activity, * Cystoseira*

## Abstract

Seaweeds have caused an emerging interest in the biomedical area, mainly due to their contents of bioactive substances which show great potential as anti-inflammatory, anti-microbial, anti-viral and anti-tumoral drugs. Despite the diversity in quality and quantity of the Mediterranean Tunisian coast flora, with its large contains of marine organisms and seaweeds, most of them have not yet been investigated for pharmacological and biological activities. Antioxidant, anti-inflammatory and antiproliferative effects of the aqueous extracts (AQ) of three brown seaweed respectively*,* *Cystoseira crinita (*AQ-*C*_cri_),* Cystoseira sedoides *(AQ-*C*_sed_) and* Cystoseira compressa *(AQ-*C*_com_) were investigated. Antioxidant activity was evaluated using the DPPH assay. Total phenolic contents were measured using Folin–Ciocalteu method. The anti-inflammatory activity of these extracts was determined *in-vivo*, using carrageenan induced rat paw oedema assay. The antiproliferative activity was studied on normal cells (MDCK and rat fibroblast) and cancer (A549, MCF7 and HCT15) cell lines by the ability of the cells to metabolically reduce MTT formazan dyes, in comparison to a reference drug the *Cisplatin*. Results demonstrated that AQ-*C*_cri_, AQ-*C*_sed_ and AQ-*C*_com_ extracts exhibited significant radical scavenging activity. AQ-*C*_com_ extract had the highest total phenolic content. AQ-*C*_cri_, AQ-*C*_sed_ and AQ-*C*_com_ extracts exhibited significant anti-inflammatory activity in a dose dependent manner by comparison to reference drugs. Moreover, AQ-*C*_cri_, AQ-*C*_sed_ and AQ-*C*_com_ extracts showed an important antiproliferative activity against both Human tumor cell lines HCT15 and MCF7. These pharmacological efficacies of these AQ- extracts of* Cystoseira* were positively correlated with their total phenol content and their good antioxidant activity. The purification and the determination of chemical structures of compounds of these active aqueous extracts are under investigation. It could have a promising role in the future medicine and nutrition when used as drug or food additive.

## Introduction

Reactive oxygen species (ROS), such as superoxide radical and hydroxyl radical, are generated in many redox processes. ROS can easily react with other molecules, such as protein, Deoxyribonucleic acid (DNA) and lipids (1) and induce oxidative damage to biomolecules. This damage may cause ageing, heart disease, stroke, arteriosclerosis, diabetes, cancer, inflammation and other many diseases (2, 3). In pathological conditions, ROS are over produced and result in lipid peroxidation and oxidative stress. In recent years, one of the areas which have attracted a great deal of attention is antioxidants in the control and prevention of those diseases in which oxidative damage has been implicated. Therefore, new interest has been developed to search natural and safe antioxidative agents from marine sources. Documented antioxidant activity would elevate their value in the human diet as food and pharmaceutical supplements (4). More recent reports revealed seaweeds to be a rich source of antioxidant compounds (5).

Over the past decades, seaweeds or their extracts have been shown to produce a variety of compounds and some of them have been reported to possess biological activity of potential medicinal value (6,7). Seaweeds are excellent source of bioactive compounds which demonstrated a broad range of biological activities such as: anti-inflammatory, antibiotics, antiviral, cytotoxic and antimitotic activities (8,9). Among these compounds, polyphenols (10), polysaccharides (11), meroterpenoids (12) and terpenoids (13) are considered as promising bioactive molecules in the search for potential therapeutic drugs.

Seaweeds have caused an emerging interest in the biomedical area, mainly due to their contents of bioactive substances which show great potential as anti-inflammatory, antimicrobial, antiviral and antitumoral drugs (14, 15). Substances that currently received most attention from pharmaceutical companies or from academic researchers for drug development or for drug design include: fucoidans, a group of sulfated polysaccharides purified from brown algae, possessing a variety of pharmacologic effects, including anticancer and anti-inflammatory properties (16). Other substances biosynthesized by algae with economic impact in food science and in human health include carotenoids, natural pigments, used as antioxidant compounds reducing the incidence of many diseases, especially those mediated by light (17). 

Many compounds such as terpenoids, alkaloids and steroids have been isolated from different species of the Mediterranean brown algae of the genus *Cystoseira *but few studies on pharmacological properties of these compounds have been published (18, 19). Despite the diversity in quality and quantity of the Mediterranean Tunisian coast flora, with its large contains of marine organisms and seaweeds, most of them have not yet been investigated for pharmacological and biological activities (20,21,22,23). Therefore, the objective of this research was to screen and evaluate antioxidant, antiproliferative and anti-inflammatory activities in aqueous extracts from *Cystoseira crinita* (AQ-*C*_cri_)*, Cystoseira sedoides* (AQ-*C*_sed_)* and Cystoseira compressa *(AQ-*C*_com_).

## Experimental

*Biological materials*

*C. crinita*; *C. sedoides* and *C. compressa *are brown algae which were collected from the Mediterranean Tunisia coasts in various areas of the coastal region of Monastir, in June 2008, at a depth between 1 and 5 m. After collection, the seaweeds were rinsed with fresh water to remove associated debris and epiphytes. The cleaned material was then air dried to dryness in the shade at 30 °C. The dried samples were finely powdered and stored at – 20 °C until use. Identification of specimens was carried out in the National Institute of Marine Sciences and Technologies (Salamboo, Tunisia). A voucher specimen has been deposited in the Department of Pharmacology, Monastir University.

*Chemicals and reagents*

Carrageenan (BDH Chemicals Ltd Poole England), Acetylsalicylate of Lysine (ASL), Dimethylsulfoxide (DMSO), Dulbecco’s modified Eagle’s minimum essential medium (DMEM), Fetal Bovine Serum (FBS), 3-(4, 5-Dimethylthiazol-2-yl)-2, 5-diphenyl-tetrazolium bromide (MTT), Penicillin and Streptomycin, were purchased from Sigma Chemical (Berlin, Germany). The following chemicals, used for antioxidant activity, were purchased from Sigma-Aldrich. Chemical Co (St. Louis, MO, USA): DPPH (2, 2- diphenyl-1- picrylhydrazyl), Folin- Ciocalteu reagent, Gallic acid (GA), Trolox (6-hydroxy-2, 5, 7, 8-tetramethylchroman-2-carboxylic acid) were obtained from Sigma Chemical. 

*Preparation of extracts*

Each Seaweed sample (400 g wet weight) was cut into small pieces mixed with a blender. Finely powdered algal material were packed in small bags (5 x 10 cm) of Whatman filter paper # 1 and all bags were sealed and macerated with water at room temperature during 24 h. The mixture was then centrifuged at 5,000 rpm for 10 min and the surnageant was filtered (Whatman No 1) to remove debris. The macerate was lyophilized during 3 days using a laboratory freeze dryer until obtaining the crude aqueous extract which was stored at 4 °C, before use for experiments.

*Antioxidant activity (AOA)*

*Total phenolic content (TPC)*

The total phenolic contents of the three aqueous extracts of the genus *Cystoseira *(AQ-*C*_cri_, AQ-*C*_sed_ and AQ-*C*_com_) were estimated by the method of Taga *et al.* (24). Briefly, 100 µL aliquot of sample were mixed with 2.0 mL of 2% Na_2_CO_3_ and allowed to stand for 2 min at room temperature. After incubation, 100 µL of 50% Folin- Ciocalteu’s phenol reagents were added, and the reaction mixture was mixed thoroughly and allowed to stand for 30 min at room temperature in the dark. Absorbance of all sample solutions was measured at 720 nm using spectrophotometer (Jenway 6505 UV/Vis). A calibration curve of gallic acid (ranging from 0.05 to 1 mg/ mL) was prepared, and TPC was standardised against Gallic acid and expressed as mg Gallic acid equivalent per gram of sample on a dry weight basis (DW). All determinations were performed in triplicate.

*DPPH radical scavenging activity*

DPPH is a chromogen-radical-containing compound that can directly react with antioxidants. DPPH has been used extensively as a free radical to evaluate reducing substances and is a useful reagent for investigating the free radical scavenging. 

When the DPPH radical is scavenged by antioxidants through the donation of hydrogen to form a stable DPPH-H molecule, the colour is changed from purple to yellow. DPPH radical scavenging activity of the three aqueous extracts of the genus *Cystoseira *(AQ-*C*_cri_, AQ-*C*_sed_ and AQ-*C*_com_) was determined according to the method of Kim (25). Each sample stock solution (1 mg/ mL) was diluted to final concentrations of 500, 250, 100, 50 and 10 (µg/ mL) in ethanol. 

A total of 0.5 mL of 30 mM DPPH ethanol solution was added to 0.5 mL of sample solution at different concentrations and allowed to react at room temperature. After 30 min, the absorbance (A) was measured at 520 nm. The ability to scavenge the DPPH radical was calculated using the following equation: 

Radical Scavenging capacity (RSC, %) = 1- [(A _sample_ – A _sample blank_) / A _control_] x 100. 

Where the A _control _is the absorbance of the control (DPPH solution without sample), the A _sample _is the absorbance of the test sample (DPPH solution plus test sample), and the A _sample blank _is the absorbance of the sample only (sample without DPPH solution). Synthetic antioxidant, Trolox was used as positive control. Concentration of extract which required reducing DPPH radicals by 50% (IC_50_) was calculated by linear regression of plots, where the abscissa represented the concentration of tested marine algae extracts and the ordinate the average percent of scavenging capacity from three replicates. DPPH was expressed in terms of Trolox Equivalent Antioxidant Capacity (TEAC) which was calculated based on its concentration of extract required to reduce DPPH radicals by 50% (IC_50_), as follows: 

TEAC (mg Trolox/ 100 g) = IC_50 (Trolox)_ / IC_50 sample_ x 100.

*Ferric Reducing Antioxidant Power (FRAP)*

Ferric reducing power of the three aqueous extracts (AQ-*C*_cri_, AQ-*C*_sed_ and AQ-*C*_com_) was determined by the method of Oyaizu (26) (Oyaizu, 1986). Briefly, 1.0 mL of each sample dissolved in distilled water was mixed with 2.5 mL of phosphate buffer (0.2 M, pH 6.6) and 2.5 mL potassium Ferricyanide (1.0%). Reaction mixture was incubated for 20 min at 50 °C. After incubation, 2.5 mL of trichloacetic acid (10%) was added, and the mixture was centrifuged for 10 min. Finally, 2.5 mL of the upper layer were mixed with 2.5 mL of distilled water and 0.5 mL of FeCl_3_ (0.1%). The solution was incubated at ambient temperature for 30 min for colour development. Absorbance of all the sample solutions was measured at 700 nm, and compared to a Gallic acid calibration curve. The data were presented as Gallic acid equivalent per gram of seaweed material (GAE/ g). A greater value of GAE related to greater reducing power of the sample.

*Anti-inflammatory activity*

Animals 

Male adult Wistar rats weighing 150 - 170 g were obtained from Pasteur Institute (Tunis, Tunisia). They were housed in polypropylene cages and were left for 2 weeks for acclimatization to animal room maintained under controlled conditions: (a 12 h light–dark cycle at 22 ± 2 ^◦^C), on standard pellet diet and water ad libitum. Before the day of assay, wistar rats were fasted overnight with the free access to water.

Animal experiments are conducted in full compliance with local, national, ethical, and regulatory principles and local licensing regulations. Housing conditions and *in-vivo* experiments were approved according to the guidelines established by the European Union on Animal Care (CFE Council (86/ 609)). The rats were used only for the anti-inflammatory evaluation of the extracts testing. 

*Carrageenan Induced Rat Paw Oedema*

Wistar rats were divided into groups of six animals. Oedema was induced by injecting 0.05 mL of 1% carrageenan subcutaneously into the sub-plantar region of the left hind paw (27). 

AQ-*C*_cri_, AQ-*C*_sed_ and AQ-*C*_com_ were administered intraperitoneally (*i.p.*) (Doses 25 or 50 mg/Kg) and were dissolved in Saline water.

The control group received the vehicle (Saline water without the extract) (2.5 mL/ Kg, *i.p.*). The reference group received acetylsalicylic of lysine (ASL, 300 mg/Kg, *i.p.*) or Dexamethasone (1 mg/Kg, *i.p.*). 

All drugs were administrated 30 min before the injection of carrageenan. Measurement of paw size was done by means of volume displacement technique using plethysmometer (Ugo Basile no.7140) immediately before carrageenan injection and 1, 2, 3, 4 and 5 h after carrageenan injection. Percentages of inhibition in our anti-inflammatory tests were obtained for each group using the following ratio: [(V_t _−V_o_) _control_− (V_t _−V_o) treated_] × 100/ (V_t_−V_o_) _control_

Where V_t_ is the average volume for each group at different hours after treatment and V_o_ is the average volume obtained for each group before any treatment. Lower and or higher doses were administered, in order to study doses dependent of the anti-inflammatory activity.

*Antiproliferative activity*

*Cell line and culture conditions*

The human tumor cell lines A549 (lung cell carcinoma), HCT15 (colon cell carcinoma) and MCF7 (breast adenocarcinoma) and normal cell lines (Mardin–Darby canine kidney (MDCK) and rat fibroblast) were purchased from the American Type Culture Collection (ATCC; Manassas, VA, USA). Freshly trypsinized cells were seeded and grown in Dulbecco’s modified Eagle’s minimum essential medium (DMEM) supplemented with 10% (v/ v) fetal bovine serum (FBS), and 1% penicillin/ streptomycin, all obtained from Biochrom AG (Berlin, Germany). They were grown on Flasks (Nunc, Denmark) at 37 ˚C in a humidified atmosphere containing 5% CO_2_. 

Cells were replicated every 2- 4 days and the medium changed once in-between. 

An aliquot of each fraction was dissolved and sterilized by 0.22µm microbiological filters (Whatman, UK) and kept at 4.0 °C before analysis. 

*Viability assay*

The potential effects on cell viability were investigated according to previously reported conditions (28, 29) using the MTT assay [3-(4, 5-dimethylthiazol-2-yl) - 2, 5-diphenyl tetrazolium bromide, Sigma-Aldrich Chimie, Saint- Quentin-Fallavier, France] as an indicator of metabolically active cells (30).

However, the development of this rapid colorimetric assay, which relies on the ability of mitochondrial dehydrogenase enzymes to convert 3, -4, 5 dimethyithiazol- 2, 5 diphenyl tetrazolium bromide (MTT) to a purple formazan precipitate, has simplified large scale screening of cells and drugs. 

The formazan crystals are dissolved and the optical density measured using a microplate reader. The use of MTT has thus become the method of choice because of its simplicity and adaptability to automation.

Concentrations ranging from 25– 250 (µg/mL) of the AQ-*C*_cri_, AQ-*C*_sed_ and AQ-*C*_com_ were prepared from the stock solutions by serial dilution in DMEM to give a volume of 200 µL in each well of a microplate reader (96- well). 

The final concentration of DMSO in the culture medium was maintained at 1% (v/v) to avoid toxicity of the solvent. A known number of A549, HCT15 or MCF7 cells (10^3^) were transferred into 96- well plates (Nunc, Denmark) in a volume of 200 µL of culture medium and incubated for 24 h before addition of test compounds. 

After 24 h, Cells were exposed at 37 ˚C to known concentrations of the different aqueous extracts to be tested. After drug exposure, cells were washed with phosphate-buffered saline (PBS) and then reincubated in fresh culture medium for a further 48 h, then the culture medium was removed and 200 µL of MTT reagent (diluted in culture medium, 0.5 mg/ mL) was added. 

Following incubation for 4 h, the MTT medium was removed and DMSO (200 µL) was added to dissolve the formazan crystals. Absorbance values were measured with a microplate reader (Bio Tek EL 340, USA) using a test wavelength of 570 nm and a reference wavelength of 630 nm. 

Results were evaluated by comparing the absorbance of the treated cells with the absorbance of wells containing cell treated by the solvent control. Conventionally, cell viability was estimated to be 100% in the solvent control.

All experiments were performed at least twice in triplicate. The concentration of substance required for 50% growth inhibition (IC_50_ value) was estimated as that resulting in 50% decrease in absorbance as compared to control incubated simultaneously without test substances.

*Data and Statistical analysis*

## Results

*Evaluation of the antioxidant activity*

*Total phenolic content*

The total phenolic content of the aqueous extracts (AQ-*C*_cri_, AQ-*C*_sed_ and AQ-*C*_com_) were measured according to Folin- Ciocalteu method. The Folin– Ciocalteu regent determines total phenols, producing blue colour by reducing yellow hetero polyphosphate molybdate tungstate anions. The total phenolic contents varied widely in the aqueous extracts (AQ-*C*_cri_, AQ-*C*_sed_ and AQ-*C*_com_) and ranged from 50.3 to 61.0 mg GAE/g dried sample (Table 1).

**Table 1:** Total phenolic content (TPC) and antioxidant activities, of aqueous extracts from three brown seaweeds; belonging to the genus *Cystoseira, *respectively: *C. crinita* (AQ-*C*_cri_), *C. sedoides* (AQ- *C*_sed_) and *C. compressa* (AQ- *C*_com_).

**Samples**		**TPC ** **(mg GAE/g ** **dried sample)**	**Antioxidant Activity (AOA)**		
			**DPPH**		**FRAP** **(mg GAE/g ** **dried sample)**
			**IC**_50_ **(µg/mL)**	**TEAC** **(mg Trolox/100g dried sample)**	
	AQ-*C*_cri_	56.5 ± 0.4	20.0 ± 0.5	450.0 ± 0.3	0.9 ± 0.5
	AQ-*C*_sed_	50.3 ± 0.1	75.0 ± 0.8	120.0 ± 0.5	0.7 ± 0.4
	AQ-*C*_com_	61.0 ± 0.3	12.0 ± 0.7	750.0 ± 0.4	2.6 ± 0.1
Trolox			90.0 0.2		

Results are expressed as means SD (*n *= 3).

TPC: Total phenolic contents; DPPH was expressed in terms of Trolox Equivalent. TEAC: Trolox Equivalent Antioxidant Capacity was calculated based on its concentration of extract which required reducing DPPH radicals by 50 % (IC_50_) as follows: TEAC (mg Trolox/ 100 g) = IC_50_ ( Trolox) / IC_50_ sample x 100.

FRAP: Ferric Reducing Antioxidant Power.

The highest levels of the total phenolic contents were found in the AQ-*C*_com_ extract with value 61.0 mg GAE/g dried sample. Whereas, in the AQ-*C*_cri_ and AQ-*C*_sed_ extracts TPC were with towards values 56.5 and 50.3 mg GAE/g dried sample, respectively. In fact, the total phenolic contents of the aqueous extracts decreased in the following order AQ-C_com _> AQ-C_cri_ >AQ-C_sed._

*DPPH radical scavenging activity*

The free radical scavenging activity was measured using the 1, 1-Diphenyl-2-picrylhydrazyl free radical (DPPH), which is a stable free radical and in the presence of the total extract it was scavenged.

The antioxidant activity was defined as the mean of free radical scavenging capacity. So we have examined the antioxidant effect of the aqueous extracts (AQ-*C*_cri_, AQ-*C*_sed_ and AQ-*C*_com_) by DPPH radical scavenging activity. These aqueous extracts were able to reduce the stable radical DPPH to the yellow coloured diphenyl prilhydrazine and the IC_50_ values were calculated and are presented in Table 1. 

The AQ-*C*_com_ and AQ-*C*_cri_ extracts exhibited excellent DPPH radical scavenging activity, with an IC_50 _value of 12 µg/mL and 20 µg/mL, respectively.

Whereas, the AQ-*C*_sed_ extracts exhibited less DPPH radical scavenging activity with an IC_50 _value of 75 µg/mL, respectively. However, the scavenging effects of these aqueous extracts (AQ-*C*_cri_, AQ-*C*_sed_ and AQ-*C*_com_) decreased in the order of AQ-*C*_com_ > AQ-*C*_cri_ >AQ-*C*_sed_. These scavenging activities were found significantly similar to Trolox (90 ± 0.2 µg/mL), under the same experimental conditions.

In the other hand, a correlation was found between the TPC and IC_50_, when the TPC was high, the IC_50 _was low and results in high level of TEAC (Trolox equivalent antioxidant capacity). This is due to the high level amount of phenolic constituents present in these aqueous extracts (AQ-*C*_cri_, AQ-*C*_sed_ and AQ-*C*_com_) which were able of functioning as free radical scavengers. 

*Ferric- reducing antioxidant power (FRAP)*

Table 1 shows greater differences in total antioxidant capacity measured by Ferric- Reducing Antioxidant Power (FRAP) method, by comparison of the three aqueous extracts. FRAP values were found within the range (0.7 and 2.6 mg GAE/g dried sample). The AQ-*C*_com_ extract has the major ability to reduce Fe^3+ ^following by the AQ-*C*_cri_ extract, with the respective value 2.6 and 0.9 mg GAE/g dried sample. The FRAP value of AQ-*C*_sed_ extract has the lower FRAP value 0.7 mg GAE/g dried sample. Aqueous extracts (AQ-*C*_cri_, AQ-*C*_sed_ and AQ-*C*_com_) had significant antioxidant activity toward the DPPH free radical and FRAP assay. 

*Evaluation of the anti-inflammatory activity*

The anti-inflammatory activity of the aqueous extracts (AQ-*C*_cri_, AQ-*C*_sed_ and AQ-*C*_com_) was investigated using carrageenan induced rat paw oedema model. 

Carrageenan has been widely used as an inflammagen capable to induce experimental inflammation used for the screening of compounds possessing anti-inflammatory activity. This phlogistic agent when injected locally into the rat hind paw of the control group induced a severe inflammatory reaction, discernible within 30 min and persists until the end of the time measurement. The maximum peak was observed between 3 h and 5 h after injection. 

As shown in Table 2, aqueous extracts (AQ-*C*_cri_, AQ-*C*_sed_ and AQ-*C*_com_) showed significant anti-inflammatory activity when administered intraperitoneally, in the carrageenan induced rat paw oedema test.

**Table 2:** Effect of the administration of the aqueous extracts for three brown seaweeds, from the genus *Cystoseira *respectively, *C. crinita* (AQ- *C*_cri_), *C. sedoides* (AQ- *C*_sed_) and *C. compressa* (AQ- *C*_com_) and both reference drugs (ASL) and Dexamethasone, on Carrageenan Induced Rat Paw Oedema

**Samples**	**Dose** **(mg/ kg)**	**Oedema (10** ^-2^ ** mL) (mean ± S.E.M)**			**Oedema inhibition (%)**		
		1h	3h	5h	1h	3h	5h
Control	-	23.2 ± 1.1	69.8 ± 1.4	74.2 ± 1.7	-	-	-
Acetylsalicylate of lysine (ASL)	300	21.2 ± 2.4**	27.1 ± 1.3**	30.3 ± 1.2**	8.6	61.2	59.2
	1	14.3 ± 1.5***	17.9 ± 3.6**	18.4 ± 1.4***	38.4	74.3	75.2
AQ- *Ccri*	25 50	14.5 ± 2.3**	17.8 ± 2.6**	19.3 ± 2.8**	37.5	74.5	73.9
		12.1 ± 2.9***	14.7 ± 2.8**	16.1 ± 2.5**	47.8	78.9	78.3
AQ- *C*_sed_	25 50	14.6 ± 2.6**	20.3 ± 3.6**	23.5 ± 2.7***	46.3	70.9	68.3
		13.7 ± 2.7**	15.6 ± 3.8**	17.5 ± 3.1***	49.6	77.6	76.4
AQ- *C*_com_	25	13.9 ± 2.2**	16.8 ± 2.7**	19.1 ± 3.6**	48.9	75.9	74.2
	50	10.3 ± 3.7**	12.5 ± 3.4**	14.7 ± 2.5**	62.1	82.1	80.2

The values represents the means difference of volume of paw ± S.E.M; n= 6.

** p < 0.01 and *** p < 0.001 significant from the Control.

AQ-*C*_cri_, AQ-*C*_sed_ and AQ-*C*_com_ tested at different doses (25 and 50 mg/Kg, *i.p*) exhibited, in a dose dependent manner, a significant inhibitory effect on the rat paw edema. The percentage of inhibition of oedema, 3 h after carrageenan injection ranged from 74.5% to 78.9% for the AQ-*C*_cri_, 70.9% to 77.6% for the AQ-*C*_sed_ and from 75.9% to 82.1%, for the AQ-*C*_com_. 

Where as the reference drug (ASL, 300 mg/Kg, *i.p*) produced 61.2 % of inhibition. However, the Dexamethasone (1 mg/Kg) showed 74.3% of inhibition of oedema at the third time. Compared to the efficacy of the reference steroidal anti-inflammatory drug Dexamethasone (used at 1 mg/Kg), the maximal inhibition was similar to those induced by AQ-*C*_cri_, AQ-*C*_sed_ and AQ-*C*_com_ at the dose of 50 mg/Kg. However, the activity profiles of these aqueous extracts differ from that of Acetylsalicylate of Lysine (300 mg/Kg), since this non-steroidal anti-inflammatory drug (NSAID) induced an inhibitory effect close to the maximal activity.

*Evaluation of the antiproliferative activity*

Results presented in Table 3, showed that the AQ-*C*_cri_ possess lower IC_50_ compared to that of the AQ-*C*_com_ on all five normal and cancer cell lines (P < 0.01). But IC_50 _of the AQ-*C*_cri_ was significantly higher than that for the AQ-*C*_sed_ and *Cisplatin* on all five normal and cancer cell lines (p < 0.01). 

**Table 3:** *In* *-* *vitro* growth inhibitory activity of three aqueous extracts from the genus* Cystoseira* respectively, *C. crinita* (AQ-*C*_cri_), *C. sedoides* (AQ-*C*_sed_) and *C. compressa* (AQ-*C*_comp_) against three human tumor cell lines A549 (lung cell carcinoma), HCT15 (colon cell carcinoma), MCF7 (breast adenocarcinoma) and two normal cell lines MDCK (Mardin–Darby canine kidney) and rat fibroblast

**Samples**	**Cancer cell lines**			**Normal cell lines**	
	A549 (IC_50_^a ^± S.D)**	HCT15 (IC_50_^a ^± S.D)**	MCF7 (IC_50_^a ^± S.D)**	MDCK (IC_50_^ a^ ± S.D)**	Fibroblast (IC_50_^ a ^ ± S.D)**
AQ-*C*_cri_	49.5 ± 0.10	26.4 ±0.30	17.9 ± 0.60	192.4 ± 0.25	182.6 ± 0.12
AQ-*C*_sed_	42.1 ± 0.40	10.5 ± 0.20	25.7 ± 0.90	190.2 ± 0.32	170.1 ± 0.19
AQ-*C*_comp_	90.3 ± 0.50	20.3 ± 0.10	29.5 ± 0.40	510.5 ± 0.27	450.3 ± 0.24
*Cisplatin*	1.5 ± 0.77	1.7 ± 0.13	1.9 ± 0.05	3.4 ± 0.11	2.5 ± 0.21

IC50: 50 percent inhibition of cell growth.^ a ^µg/ mL, *P < 0.05, **P< 0.01

The lower IC_50_ represents the higher potency of a compound to inhibit the growth of cells and cause toxicity and death of cells. Comparison of the evaluated IC_50 _of the AQ-Ccri AQ-Ccom AQ-Csed extracts with that of Cisplatin, on normal and cancer cell lines (Table 3) showed that the IC50 of the tested samples on the five cell lines increased in the following rank order: *Cisplatin *< AQ-*C*_sed_ < AQ-*C*_cri_ < AQ-*C*_com_. The IC_50 _of *Cisplatin* on the five cell lines decreased in the following rank order of cells: MDCK> fibroblast > MCF7 > HCT15 > A549.However, the lowest and highest IC_50_ values were related to *Cisplatin* and AQ-*C*_com_*. *Also, the IC_50_ of AQ-*C*_com_ on the five cell lines decreased in the following rank order of cells: MDCK > fibroblast > A549 > MCF7 > HCT15.

On the other hand, the IC_50_ of AQ-*C*_sed_ extract on the five tested cell lines increased in the following order of cells: MDCK < fibroblast < A549 < MCF7 < HCT15. As a result, the highest and lowest cytotoxicity AQ-*C*_sed_ was related to HCT15 (IC_50_ = 10 µg/mL) and MDCK (IC_50_ = 190 µg/mL) cell lines. 

The IC_50_ of AQ-*C*_com_ on the five cell lines increased in the following rank order of cells: MDCK < fibroblast < A549 < MCF7 < HCT15. However, the highest and lowest cytotoxicity of the AQ-*C*_com_ was related to A549 (IC_50_ = 90 µg/mL) and MDCK (IC_50_ = 510 µg/mL) cell lines. 

Treatment with the aqueous extracts (AQ-*C*_cri_, AQ-*C*_sed_ and AQ-*C*_com_) induced concentration dependent inhibition on the growth of cancer cells lines.

After 2 days treatment, no-microscopically visible alteration on normal cell was observed even at 250 µg/mL. In addition, viability assay showed no=destruction of cell layer. 

**Figure 1:**
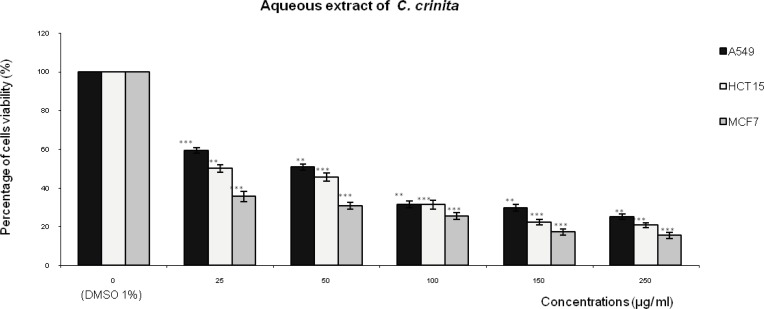
Effect of the aqueous extract of *Cystoseira crinita *(AQ- *C*_cri_) on the viability of three human tumor cells lines (A549: lung cell carcinoma; HCT15: colon cell carcinoma and MCF7: breast adenocarcinoma). Expressed as (%) of cell viability to the control. Statistical significance is based on the difference when compared with the cells without treating extract (**p < 0.01, ***p < 0.001).

**Figure 2:**
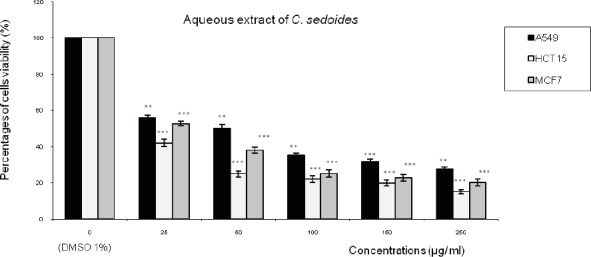
Effect of the aqueous extract of *Cystoseira sedoides *(AQ-*C*_sed_) on the viability of three human tumor cells lines (A549: lung cell carcinoma; HCT15: colon cell carcinoma and MCF7: breast adenocarcinoma). Expressed as (%) of cell viability to the control.

**Figure 3:**
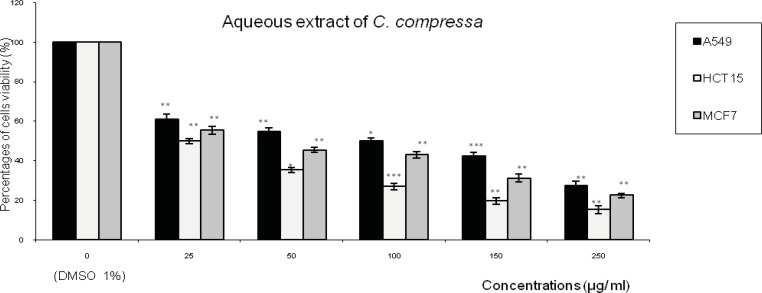
Effect of the aqueous extract of *Cystoseira compressa* (AQ-*C*_com_) on the viability of three human tumor cells lines (A549: lung cell carcinoma; HCT15: colon cell carcinoma and MCF7: breast adenocarcinoma). Expressed as (%) of cell viability to the control. Values are means ± SD (n= 3). Statistical significance is based on the difference when compared with the cells without treating extracts (**p < 0.01, ***p < 0.001).

Figures 1,2,3 represent respectively the concentration effectiveness of (AQ-*C*_cri_, AQ-*C*_sed_ and AQ-*C*_com_) on viability cells lines (A549, HCT15 and MCF7) using MTT assay. A mitochondrial enzyme in living cells, succinate-dehydrogenase, cleaves the tetrazolium ring, converting the MTT to an insoluble purple formazan. 

Therefore, the amount of formazan produced is directly proportional to the number of viable cells. Five different concentrations of each extract (25, 50, 100, 150 and 250 µg/mL of each aqueous extract) were applied. AQ-*C*_cri_, AQ-*C*_sed_ and AQ-*C*_com_) inhibited HCT15 and MCF7 cancer cell growth in a concentration dependent manner. 

Treatment with the AQ-*Ccri* at concentrations ranging from 25 to 250 µg/mL (Figure 1) produced important cell growth inhibition in, MCF7 and HCT15 cancer cell lines, from 64% to 74% and from 49% to 79%, respectively. Whereas, the inhibition effect on cells growth of A549 cells was ranging from 40% to 74%. 

Treatment with AQ-*Csed* in levels of cell growth inhibition ranging from: 57% to 84 % in HCT15 cells followed by 47% to 79% in MCF7 cells and 44% to 72% in A549 (Figure 2). The level of cell growth inhibition observed with AQ-*C*_com_ at concentrations ranging from 25 to 250 µg/mL for 24 h (Figure 3) was from 50 to 84% in HCT15 cells followed by 44% to 77% in MCF7 cells and 39% to 72% in A549 cells.

Statistical significance is based on the difference when compared with the cells without treating extract (**p < 0.01, ***p < 0.001).

## Discussion

The present study was conducted to evaluate the antioxidant, the anti-inflammatory and the antiproliferative activities of of the aqueous extracts (AQ-*C*_cri_, AQ-*C*_sed_ and AQ-*C*_com_).

AQ-*C*_cri_, AQ-*C*_sed_ and AQ-*C*_com _showed remarkable antioxidant activities. In addition, these aqueous extracts showed higher DPPH radical and an important ferric reducing power activity. Different findings of the amount of the total phenolic contents in brown seaweeds have been reported: from 1.23 to 3.28 mg GAE/g of *Stypocaulon scoparium *extracts (31); from 2.78 to 26 mg GAE/g of organic fractions from three selected brown seaweeds of India (32). Therefore, the TPC compounds in the aqueous extracts might explain their high antioxidant activities. The positive correlation between total phenolic contents of alga and its antioxidant activity is well documented (33). 

Many researchers have investigated the antioxidant factors of seaweeds. Several compounds were identified as antioxidant, including protective enzymes (34), ascorbic acid (35), lipophilic antioxidants (36), phlorotannins (37) and catechins (38). 

Increasing scientific evidence shows that polyphenols are good antioxidants and are effective in preventing cardiovascular and inflammatory diseases, and can also be used as chemopreventing agents for cancer. These molecules might act as cancer-blocking agents, preventing initiation of the carcinogenic process and as cancer-suppressing agents, inhibiting cancer promotion and progression (39).

In addition, free radicals play a crucial role in the pathophysiology of human diseases such as inflammation, cancer and neurodegenerative diseases, and considerable attention has been focused to protect against these diseases. 

Many antioxidants are being identified as anticarcinogens (40). Yuan and Walsh (41) have proved antioxidant and antiproliferative activities of extracts from a variety of marine algae on human cervical adenocarcinoma cells. So, there should be a close correlation between the content of phenolic compounds and antioxidant and anticancer activities (42).

Findings of this study revealed aqueous extracts*:* AQ-*C*_cri_, AQ-*C*_sed_ and AQ-*C*_com_ (25 and 50 mg/Kg) produced a marked inhibition on carrageenan-induced rat paw oedema when compared to Dexamethasone (1 mg/Kg), which was used as a standard anti-inflammatory drug. However the ASL (300 mg/Kg) was less potent than this reference drug as well for the different evaluated aqueous extracts. It is known that the oedema induced by carrageenan involves different phases with the participation of different chemical mediators, such as histamine, serotonine, kinine prostanoids and leucotriens (43). The anti-inflammatory effect exhibited by the aqueous extracts of these brown seaweeds suggest that these aqueous extracts with their actives principles could possibly interfere with some of the mediators, by inhibiting their productions or antagonize their actions (44). The anti-inflammatory potencies of the aqueous extracts AQ-*C*_cri_, AQ-*C*_sed_ and AQ-*C*_com_ may be due in part to the important quantity of phenolic compounds contained in these extracts: 56.5; 50.3 and 61.1 mg GAE/g dried sample, respectively. 

Chemical and biological investigations indicated that the main substances biosynthesized by brown algae with anti-inflammatory potential include: sulfated polysaccharides, phlororotanins, carotenoids and it is possible that some of these bioactive compounds present in the aqueous extracts are responsible at least in part for the anti-inflammatory activity (45). 

Indeed, several pharmacological studies reported that the sulfated polysaccharides, fucoidans, present in brown algae possess anti-inflammatory properties. These compounds are a potent selectin blocker and have been used experimentally to prevent inflammatory damage after ischemic events (46). Fucoidans have been shown, also, to inhibit phospholipase A2, an important enzyme in the inflammatory cascade (47) and appear to inhibit the functions of macrophages, a predominant source of pro- inflammatory factors (48). Kim *et al.* (49) reported that ethanolic extract of the brown algae, *Ishige okamurae*, was effective in inhibiting the production of 22 inflammatory mediators, such as TNF-α, Il-1β, Il-6 and PG-E2 in Raw 264.7 macrophage cells and by inactivation of NF-χB transcription factor in macrophages stimulated by lipopolysaccharide. Myers* et al.* (50) showed, in an open label combined phase I and phase II pilote scale study in osteoarthritis of Knee, that formulation, containing a blend of extracts from three different species of brown algae, when taken orally by patients over twelve weeks decreased the symptoms of osteoarthritis in a dose dependent manner. Moreover, brown seaweed extracts, have been demonstrated to contain also phlorotanins, polyphenolic compounds with anti-inflammatory activity. Investigations conducted by Sugiura *et al*. (51) demonstrated that MeOH/CHCl_3 _extract from *E. arborea *inhibited inflammatory mediators (histamine and eicosanoids: LTB4, PGD2) release from RBL cells and that phlorotanins and methanol/chloroform extract inhibited activities of enzymes (phospholipase A2, cyclooxygenase A2, lipoxygense) involved in eicosanoids synthesis in the arachidonate cascade. In addition to the polar components (fucoidans, and phlorotanins), brown algae produced also non polar components, such as carotenoids, with anti-inflammatory potential. Recently, it has been claimed that fucoxanthin, one of the most abundant carotenoids isolated from brown algae, exert anti-inflammatory effect via inhibitory effect of nitric oxide production, in lipopolysaccharide induced Raw 264.7 macrophage cells (52). Considering this and previous studies, we can deduce that the anti-inflammatory activities exerted by the aqueous extracts is probably due to the synergistic effects of polar and non polar component produced by this brown algae to reduce experimental inflammation induced by carrageenan. According to the results obtained on the IC_50_ of AQ-*C*_cri_, AQ-*C*_sed_ and AQ-*C*_com_ and *Cisplatin*, as aqueous extracts of these brown seaweed and the chemical control positive compounds; respectively on normal cell lines were higher than that on cancer cell lines. Both AQ-*C*_cri_ and AQ-*C*_sed _ exhibited important antiproliferative effects against all cell lines. 

Chemical and biological investigations indicated that the main substances biosynthesized by brown algae with antiproliferative and antitumor potential include: sulfated polysaccharides, phlorotannins, terpenes and the possibility that some of these bioactive compounds present in the aqueous extracts are responsible in part for the anti-proliferative activity. Indeed, several studies reported that the sulfated polysaccharides, fucoidans, present in brown algae have antiproliferative activity in cancer cell lines *in-vitro* as well as inhibitory activity of tumor grown in mice and they have antimetastatic activity by blocking the interactions between cancer cell lines and the basement membrane (53). Alekseyenko *et al.* (54) reported that fucoidans, isolated from the brown algae *Fucus evanescens *and administrated, at a dose of 10 and 25 mg/Kg, to C57B1/ 6 mice with transplanted lewis lung adenocarcinoma, potentiated the anti-metastatic and anti-tumor activities of cyclophosphamide, respectively. It has been shown also that these sulfated polysaccharides, inhibited the growth of human gastric adenocarcinoma cells by inducing autophagy as well as apoptosis (17). The mechanisms through which fucoidans exert their antiproliferative effects are not completely understood because of their remarkable structural diversity which entails multiple interactions (55).

In addition to its anti-inflammatory activity, phlorotannins and polyphenolic compounds of brown seaweeds possess also antiproliferative properties. Indeed, Kong *et al.* (56) reported that isolated phloroglucinol derivatives from *Ecklonia cava, *exerted a higher antiproliferative activity in MCF7 human cancer cells and apoptosis in a concentration related manner. Extracts from brown algae, *Laminaria japonica*, has been shown to induce inhibition of human hepato-cellular carcinoma cells (BEL7402) and murine leukemic cells (P388) using MTT assay. The IC_50_ of these extract were > 120 µg/mL. 

The anti-proliferative activity of this extract is associated with the total phlorotanin algal content (57). In addition to the polar components (fucoidans, and phlorotanins), brown algae produced also non polar components, such as terpenes, with anti-proliferative potential: the hydroquinone diterpene mediterraneol, from *Cystoseira mediterranea* is an inhibitor of mitotic cell divisions (58) and the meroterpene, usneoidone E and Z, from *Cystoseira usneoides*, have antitumor properties (59,60). The isolated diterpenes from methanol extract of *Padina pavonia*, collected from the red sea, showed antitumor activities against lung carcinoma (H460) and liver carcinoma (HepG2) human cell lines (61). Some authors reported that *C. myrica*, collected in the Gulf of Suez, yielded four hydroazulene diterpenes, dictyone acetate, dictyol F monoacetate, isodictytriol monoacetate and cystoseirol monoacetate (62). These reports suggest that diterpenes compounds could be responsible for the antiproliferative activity measured in the brown seaweed of the genus *Cystoseira* collected in our study.

Some others scientific (63,64) reported that there is a close relationship between inflammation and cancer; in which tumor promoters recruit inflammatory cells to the application site and cancer development may also act by aggravating inflammation in the tissue and vice versa and that inflammatory cells are capable of inducing genotoxic effects (65). 

## Conclusion

This study revealed that AQ-*C*_cri_, AQ-*C*_sed_ and AQ-*C*_com_ provide an interesting anti-inflammatory activity associated with significant antiproliferative activity and these activities of aqueous extracts of the genus *Cystoseira* which are associated with the total phenolic algal contents and strong antioxidant activity. 

These findings confirm other results which have been reported recently and related to different species of brown algae and their bioactive compounds. Therefore further chemical investigations are needed to determine in different aqueous extracts; polar components such as sulphated polysaccharides and non polar such as terpenes and carotenoides responsible of anti-inflammatory and antiproliferative activities. The AQ-*C*_cri_, AQ-*C*_sed_ and AQ-*C*_com_ extracts can be used as easily accessible source of natural antioxidants and as a possible food supplement or in pharmaceutical industry. 
